# Correlation analysis between the magnetic resonance imaging characteristics of osteoporotic vertebral compression fractures and the efficacy of percutaneous vertebroplasty: a prospective cohort study

**DOI:** 10.1186/s12891-018-2040-8

**Published:** 2018-04-12

**Authors:** Wei Xu, Silian Wang, Chao Chen, Yifan Li, Yunhan Ji, Xiaodong Zhu, Zhikun Li

**Affiliations:** 1 Department of Orthopedics, Tongren Hospital, Shanghai Jiao Tong University School of Medicine, 1111 XianXia Road, Shanghai, 200336 People’s Republic of China; 2 Department of Radiology, Tongren Hospital, Shanghai Jiao Tong University School of Medicine, 1111 XianXia Road, Shanghai, 200336 People’s Republic of China; 3 Medical Services Section, Tongren Hospital, Shanghai Jiao Tong University School of Medicine, 1111 XianXia Road, Shanghai, 200336 People’s Republic of China

## Abstract

**Background:**

To explore the relationship between the magnetic resonance imaging (MRI) characteristics of osteoporotic vertebral compression fractures (OVCFs) and the efficacy of percutaneous vertebroplasty (PVP).

**Methods:**

A prospective study was conducted to analyze the clinical and imaging data of 93 patients with OVCFs treated via PVP. A visual analogue scale (VAS), the Oswestry Disability Index (ODI), and the Medical Outcomes Study(MOS) 36-Item short-form health survey (SF-36) were completed before surgery as well as 1 day and 1, 6, and 12 months after surgery. In addition, postoperative complications were recorded. According to the degree and ranges of bone marrow edema on MRI, the patients were divided into three groups: the mild (group A), moderate (group B), and severe (group C) bone marrow edema groups. Pain and dysfunction scores were compared across the three groups of patients before surgery as well as 1 day and 1, 6, and 12 months after surgery.

**Results:**

The VAS, ODI, and SF-36 scores showed significant differences (*P* < 0.05) before and after surgery among the three groups. The ODI and SF-36 scores were significantly different (P < 0.05) at 1 day and 1 month after surgery among the three groups. Groups A and B showed significantly better pain relief than group C. Group B experienced better pain relief than group A. These results indicate that PVP was associated with better pain relief effects among patients with a greater extent of bone marrow edema. The edema ranges of the vertebral fractures were negatively correlated with the postoperative VAS and ODI scores 1 month after surgery, whereas the ranges were positively correlated with postoperative SF-36 scores 1 month after surgery.

**Conclusions:**

PVP is an effective treatment for OVCFs. Better outcomes were observed among patients with severe or moderate bone marrow edema rather than those with mild bone marrow edema. A greater degree of pain relief after PVP was correlated with faster recovery of the postoperative function. However, this correlation gradually became weak over time and disappeared 6 months after surgery. Therefore, PVP should be an option for early stage OVCFs, especially among patients with bone marrow edema signs on MRI.

## Background

Population aging contributes to the annual increase in the proportion of elderly people in China. To date, 88 million people are aged 65 years or over, which accounts for 7% of the total population and indicates a considerable number of elderly people. Osteoporosis is a common disease among the aged population, and it has increasingly drawn public attention due to concerns regarding population aging. Characterized by reduced bone mass, decreased bone strength, and bone microstructure degeneration, osteoporosis is a systemic disease that can cause increased bone fragility and risk of fracture [1]. The most serious consequence caused by osteoporosis is osteoporotic vertebral compression fracture (OVCF), which results in significant morbidity and decreased quality of life. The clinical manifestations of OVCFs are numerous, and the most serious symptom is severe pain [2–4]. Severe pain caused by OVCFs might not respond to conservative therapies including bed rest, the use of analgesics, and supportive devices; moreover, this pain can lead to many complications [5–7].

Percutaneous vertebroplasty (PVP) has become the gold standard to treat acute and chronic OVCFs, and it can effectively relieve the refractory pain in the middle or lower back caused by vertebral fractures [8–10]. However, a variety of factors can affect the degree of pain relief and functional recovery in individuals [11–13].

Pain relief in patients with OVCFs after PVP is associated with the duration of disease, bone density, the amount of injected bone cement, and the severity of vertebral compression. Magnetic resonance imaging (MRI) can sensitively detect the bone marrow edema caused by vertebral compression fractures, which can indicate acute or subacute fractures [14–16] and is considered as the best indication for PVP, However, the relationship between bone marrow edema and PVP was not further explained. In clinical practice, bone marrow edema signs on MRI among patients with vertebral compression fractures is a useful predictor for postoperative pain relief and functional recovery. The purpose of this study was to investigate the correlation between the MRI characteristics of OVCFs and the efficacy of PVP, the hypothesis of study is that the degree of edema predicts degree of success of injection.

## Methods

### Patient selection and grouping

In this study, we included patients with OVCFs who underwent PVP at Shanghai Tongren Hospital since January 2013. These patients signed an informed consent document before surgery. The Shanghai Tongren Hospital Ethics Committee reviewed and approved this study in January 2013 (project number: 20130111). The inclusion criteria were as follows: the diagnostic criteria for OVCFs were met; single-segment vertebral body compression fracture was confirmed; and preoperative MRI of the vertebral bodies was performed.

Compression fractures were diagnosed based on X-ray, Computed Tomography(CT), and MRI images. The diagnostic criterion for osteoporosis was a T-score of <− 2.5 in the dual-energy X-ray absorptiometry (DXA) of the lumbar spine and hip. All patients presented with pain in the lower back corresponding to the fracture site with or without anterior-thoracic or abdominal radiating pain. No symptoms of spinal cord or nerve root compression were reported. Surgical contraindications, including infection, hemorrhagic disease, and severe vertebral compression fractures, were ruled out.

**Fig. 1:**
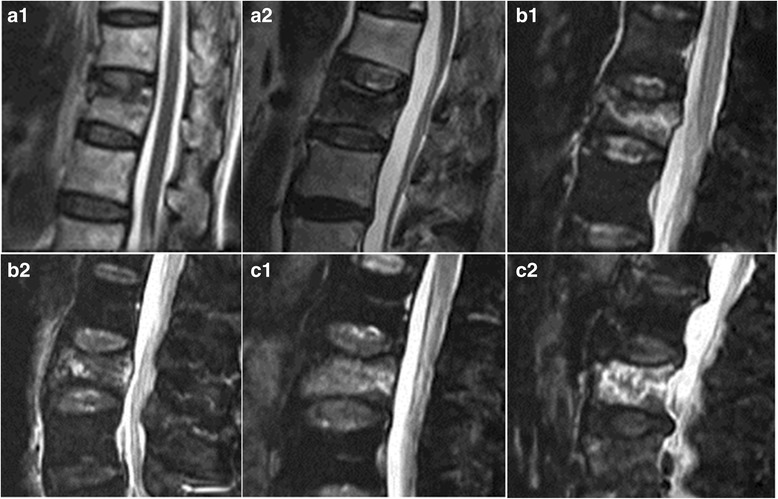
According to the degree and ranges of bone marrow edema on the MRI sagittal view, the patients were divided into mild edema(a1、a2), moderate edema(b1、b2), and severe edema(c1、c2)

MRI of the affected vertebral body was performed before surgery to identify the fracture site and distinguish new fractures from old fractures. A 1.5 T MRI scanner was used for this study. The fractures with bone marrow edema signals (i.e., low T1-weighted signals and high T2-weighted signals) were classified as newly onset fractures(usually within 2 weeks after the fracture). According to the degree and ranges of bone marrow edema on the MRI sagittal view, the patients were divided into group A (mild edema ranging from 1% to 24%), group B (moderate edema ranging from 25% to 74%), and group C (severe edema ranging from 75% to 100%), Fig. 1. Experienced radiologists completed the MRI evaluations. Percent = the area of bone marrow edema/ the area of the entire vertebral body*100%.

### Methods

#### Surgical procedure

Under general anesthesia, the patients assumed the prone position with two hands alongside their heads. The effected vertebral body was confirmed via physical examination, X-ray,CT and MRI. Usually, we estimate the position of the vertebral body by the bone marker. For example, the iliac crest is at the L4/5 level. Place the K wires in the estimated position and identify the fracture vertebra by comparison with X-ray intraoperative and X-ray,CT,MRI pre-operation. It is necessary to make full use of the bone markers in the X-ray for positioning, and sometimes it needs to use multiple kirschner wire. For example, if T12 fracture, according to T12 whether there are ribs, vertebral body compression, greater osteophyte and so on to confirm the vertebral body. If L4 fracture, according to the position of sacrum, vertebral body compression, greater osteophyte and so on to confirm the vertebral body. A digital subtraction angiography (DSA) machine was used to locate the affected vertebral body and the medial edges of the bilateral pedicles to determine the puncture approach and position angle of the puncture needle and to mark the body’s surface. The operative field was disinfected and draped. Under the fluoroscopic guidance of the DSA machine, the access needles (Stryker Inc., USA) were advanced into the anterior-middle 1/3 of the affected vertebral body along the sagittal plane. The position of the needle tip was confirmed via fluoroscopy (anteroposterior and lateral views). Standard bone cement, polymethyl methacrylate (stark, USA), was injected into the affected vertebral body until it was distributed to the posterior margin of the vertebral body or stop the injection when serious imaging complications are found. In other words, in intraoperative X-ray, a large amount of bone cement leaked outside the vertebral body. The amount of the injected bone cement was documented. The patient was required to use a waist supportive belt to help with ambulation for 2 weeks.

#### Clinical parameters

Patient age, gender, bone mineral density, amount of the injected bone cement, duration of disease, and degree of vertebral compression were documented. The degrees of vertebral compression fracture were defined as follows: A vertebral compression with less than 1/4 of the vertebral body height loss was recorded as 1; vertebral compression with 1/4 to 1/2 of the vertebral body height loss was recorded as 2; and vertebral compression with more than 1/2 of the vertebral body height loss was recorded as 3. Vertebral compression fractures of T1 through T10 were recorded as 1, T11 to L2 as 2, and L3 to L5 as 3.

Efficacy observations using a visual analogue scale (VAS), the Oswestry disability index (ODI), and the MOS item short-form health survey (SF-36) were completed before surgery as well as 1 day and 1, 6, and 12 months after surgery

- A VAS was used to assess pain severity. The VAS (0–10) categories are as follows: 0, no pain; < 3, mildly tolerable pain; 4 to 6, moderate, reluctantly tolerable pain that affects sleep quality; and 7 to 10: severe or worst possible, an intolerable pain that affects appetite and sleep quality.
- The ODI was used to evaluate the patient’s daily activity and degree of dysfunction. The ODI questionnaire contains 10 items addressing pain intensity, lifting, ability to care for oneself, ability to walk, ability to sit, sex life (if able), ability to stand, social life, sleep quality, and ability to travel. Each item has six options, representing a 5-point scale. The total was calculated and then divided by 50 to obtain the ODI score. For practical application, the scores are multiplied by 100. Zero represents no disability, and 100 denotes the maximum disability possible.
- The SF-36 was used to evaluate the patient’s physical and mental statuses. The SF-36 consists of eight scaled scores, including physical role functioning, social role functioning, physical functioning, mental health, emotional role functioning, vitality, bodily pain, and general health perceptions. Standard physical health and mental health scores are calculated based on the aforementioned eight sections.

### Statistical analyses

The data are expressed as means±standard deviations. Statistical Product and Service Solutions(SPSS) 21.0 was used for statistical analysis. Paired t-tests were used to compare differences with regard to the VAS, ODI, and SF-36 scores within the three groups (i.e., comparisons before and after treatment in each group and comparisons between the groups). Independent-samples t-tests were used to compare the difference between groups. Spearman’s rank correlation coefficient was used to analyze the correlation between the bone marrow edema signs of the fractured vertebral body and each efficacy parameter. *P* < 0.05 was considered as significant.

## Results

### General surgical data

**Fig. 2:**
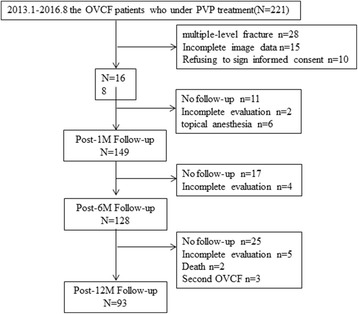
Flow chart of follow-up

As of August 2016, 93 patients were included in this study (Fig. 2), including 18 men and 75 women. The average age was 73.4 ± 10.3 years, ranging from 55 to 99 years. Their bone mineral density was − 2.9 ± 0.45. The amount of injected bone cement was 4.3 ± 0.8 mL. The duration of disease was 1.34 ± 0.561 days. Fifty-two and 44 of the 93 vertebral fractures were thoracic and lumbar vertebral fractures, respectively. The degree of vertebral compression fracture was 1.75 ± 0.747. The number of vertebral fracture segments was 1.96 ± 0.44. The aforementioned parameters did not significantly differ among the three groups (*P* > 0.05; Table 1).

**Table 1:** Paired t-tests were used to compare differences with regard to the parameters within the three groups

Parameters	Group A(*n* = 30)	Group B(*n* = 46)	Group C(*n* = 11)	*P*
Age(year)	73.9 ± 10.2	76.4 ± 10.8	71.2 ± 8.4	> 0.05
Gender(Female/Male)	21/9	20/6	19/7	> 0.05
BMD	−2.92 ± 0.46	−2.88 ± 0.42	− 2.96 ± 0.50	> 0.05
PMMA(ml)	4.2 ± 1.3	4.4 ± 1.5	3.8 ± 1.1	> 0.05
Time(day)	1.4 ± 0.563	1.31 ± 0.618	1.31 ± 0.549	> 0.05
Degree	1.70 ± 0.75	1.78 ± 0.758	1.76 ± 0.752	> 0.05
VB	1.93 ± 0.365	1.96 ± 0.469	2.0 ± 0.5	> 0.05

*BMD* bone mineral density, *PMMA*, the injection volume of PMMA in PVP operation, *Time* course of disease, *Degree* The degree of vertebral compression, *VB* Compression vertebral body

### Observation of efficacy

The VAS, ODI, and SF-36 evaluations showed that postoperative pain was reduced significantly in groups A, B, and C (*P* < 0.05).

The VAS, ODI, and SF-36 scores did not significantly differ among the three groups (*P* > 0.05) before surgery. The VAS, ODI, and SF-36 scores significantly differed (*P* < 0.05) before and after surgery among the three groups. The ODI and SF-36 scores significantly differed (*P* < 0.05) 1 day and 1 month after surgery among the three groups. The VAS, ODI, and SF-36 scores did not significantly differ (*P* > 0.05) when comparing 1 month with 6 months and when comparing 6 months with 12 months after surgery. Significant pain relief was observed in groups A and B compared with group C. Group B achieved better pain relief than group A. These results indicate that patients with moderate or severe bone marrow edema can achieve better pain relief after PVP (Table 2).

**Table 2:** Paired t-tests were used to compare differences with regard to the VAS, ODI, and SF-36 scores within the three groups

Period		Group A(*n* = 30)	Group B(*n* = 46)	Group C(*n* = 17)	*P*
Per	VAS	7.7 ± 1.3	8.4 ± 1.0	7.8 ± 1.3	> 0.05
	ODI	75.9 ± 5.1	83.1 ± 5.2	77.8 ± 4.4	> 0.05
	SF-36	24.5 ± 4.3	26.1 ± 3.5	25.2 ± 2.6	> 0.05
Post	VAS	4.0 ± 2.6	3.5 ± 2.0	3.0 ± 2.1	<0.05*
	ODI	33.4 ± 3.9	28.9 ± 3.3	23.3 ± 4.2	<0.05*
	SF-36	26.7 ± 4.3	30.2 ± 4.6	33.7 ± 5.2	<0.05*
Post-1 M	VAS	3.2 ± 1.5	3.0 ± 1.9	2.8 ± 1.5	>0.05
	ODI	26.1 ± 3.2	22.6 ± 3.0	19.5 ± 2.9	<0.05*
	SF-36	29.1 ± 3.5	35.1 ± 4.4	37.9 ± 4.8	<0.05*
Post-6 M	VAS	3.1 ± 1.5	3.0 ± 1.8	2.8 ± 1.6	>0.05
	ODI	22.2 ± 3.1	21.5 ± 2.9	21.8 ± 1.6	>0.05
	SF-36	35.3 ± 3.2	36.9 ± 3.9	35.1 ± 2.5	>0.05
Post-12 M	VAS	3.0 ± 1.2	2.9 ± 1.5	2.8 ± 1.5	>0.05
	ODI	21.2 ± 3.0	21.4 ± 2.6	21.9 ± 1.5	>0.05
	SF-36	34.3 ± 2.2	35.5 ± 3.7	35.4 ± 2.1	>0.05

Note: *P<0.05

### Correlation analysis

The edema ranges of the vertebral fractures were negatively correlated with the postoperative VAS and ODI scores 1 month after surgery; however, the ranges were positively correlated with postoperative SF-36 scores 1 month after surgery. (Table 3).

**Table 3:** The spearman’s relevance bewteen Vertebral marrow edema and VAS,ODI,SF-36

Evaluated Parameters		Correlation Coefficient	*P*
VAS	Per	−0.31	0.766
	Post	−0.522**	<0.001
	Post 1 M	0.001	0.991
	Post 6 M	0.121	0.246
	Post 12 M	0.15	0.884
ODI	Per	0.178	0.088
	Post	−0.808**	<0.001
	Post 1 M	−0.735*	<0.05
	Post 6 M	−0.135	0.196
	Post 12 M	0.112	0.283
SF-36	Per	0.086	0.414
	Post	0.802**	<0.001
	Post 1 M	0.688**	<0.05
	Post 6 M	0.002	0.987
	Post 12 M	0.071	0.500

Note:*P<0.05,**P<0.01

### Complications

The intraoperative leakage of bone cement into the anterior or posterior vertebral bodies of the three groups was noted in 5 and 1 patient(s), respectively. However, these patients were asymptomatic after surgery. After a 1-year follow-up period, no complaints (e.g., pain or progressive pain) were reported. No worsening dysfunction was evident in daily activity.

## Discussion

In general, the pathogenesis of primary OVCFs of the spine can be divided into five phases [17, 18]: (1) the occurrence of fractures and vertebral ischemic changes; (2) vertebral bone marrow edemas that are present as high T2WI on MRI, although the lesion enhancement is not shown in the enhanced scan; (3) irregular enhancement at the compressive vertebral body can be noted during early stage of fracture healing due to increased blood supply during tissue repair; (4) the degree of enhancement at the compressive vertebral body gradually decreases during the late stage of fracture healing because the increased blood supply gradually becomes normal; and (5) the compressive vertebral body is gradually replaced by adipose tissue during the chronic phase. Although the fractured vertebral body can be compressed into a “coin-like” tissue, the T1WI and T2WI signals gradually increased, especially the T1WI signal. If necrotic liquefaction is present in the fractured vertebral body, then the intensity of this vertebral body might not change during an enhanced scan. On the other hand, the mechanism of pain relief via PVP remains unclear and might involve the following aspects [14]. (1) Mechanical manipulation - The injection of bone cement can improve the biomechanical properties of the spine, fixate micro-fractures, and reduce the tiny migration of the fracture fragments, thereby reducing the stimulation to nociceptive nerve endings. Because trabecular spacing in the case of osteoporosis increases, bone cement can be filled into the entire vertebral body via increased trabecular spacing (i.e., vertebral augmentation that supports the vertebral body to effectively prevent vertebral collapse and compression fractures). (2) Thermal effect of the bone cement - The polymerization reaction of bone cement through which the peak temperature generated is between 52 and 93 °C can lead to necrosis of the surrounding tissue and the destruction of the nerve endings. Thus, pain subsides or is alleviated. (3) Chemical effects - Bone cement has its own cytotoxic effect that can kill the tumor tissue.

**Fig. 3:**
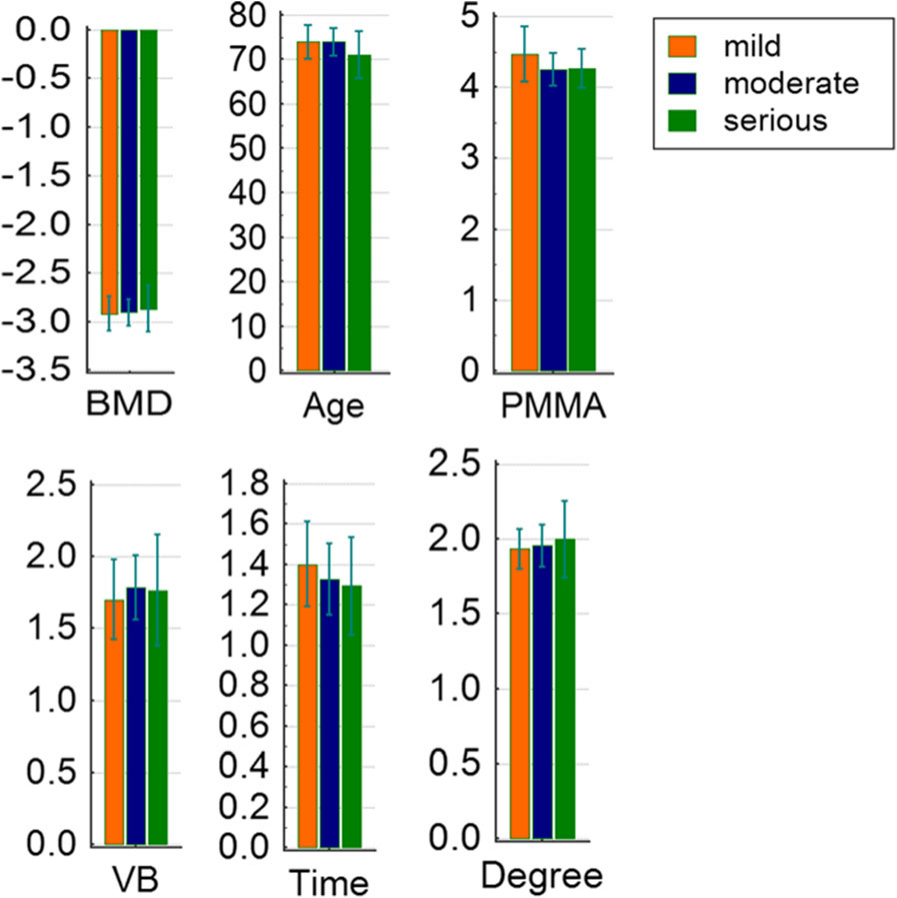
Statistical graphs of various parameter in different groups

**Fig. 4:**
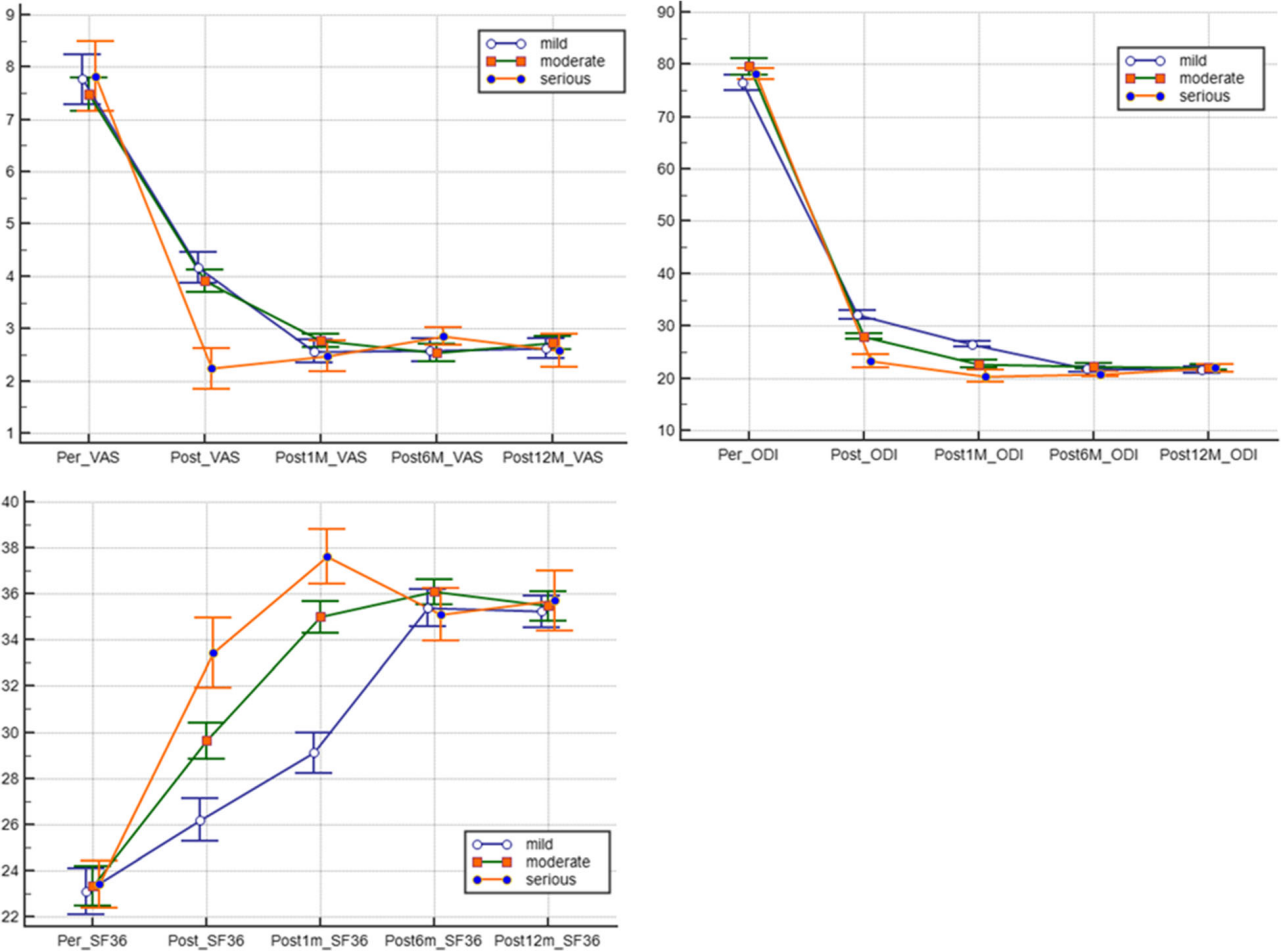
Changes in VAS,ODI,SF-36 during different group follow-up

This study divided the patients into mild, moderate, and severe groups. Patient age, sex, bone mineral density, amount of injected bone cement, duration of disease, and degree of vertebral compression fracture before surgery did not significantly differ (*P* > 0.05). The results revealed significant differences in the VAS, ODI, SF-36 scores with regard to pain severity before and after surgery among patients with different bone marrow edema signs of vertebral fractures (*P* < 0.05), indicating that PVP is effective, regardless of the extent of bone marrow edema, Fig. 3. Moreover, these results indicate that patients with moderate or severe bone marrow edema can achieve better pain relief after PVP. Nevertheless, sharp decreases in the postoperative VAS and ODI scores and a rapid increase in the SF-36 score were observed in patients with larger ranges of bone marrow edema (group C). In patients with different degrees of bone marrow edema, the difference in efficacy was significant before and immediately after surgery; however, this difference gradually became smaller. On the 6th month after surgery, no difference in efficacy was observed among the three groups (*P* < 0.05). This result demonstrates that PVP is more effective in patients with larger ranges of bone marrow edema during the early stage (within 6 months) after surgery but has a similar effect 6 months after surgery (Fig. 4).

MRI is a routine examination before PVP among patients with OVCFs. In addition to its high resolution regarding soft tissue, the skeletal muscles, or the nervous system, MRI is sensitive to the presence, magnitude, and location of bone marrow edema of the vertebral compression fracture. The changes in bone marrow signal intensity can reflect the stages of OVCFs and the phases of bone healing [16, 19]. In general, OVCFs are present with low T1WI and high T2WI signals on MRI within 1 month after fractures and tend to become normal (i.e., isointense T1WI and T2WI) after 1 month; then, they return to normal bone marrow signals after complete healing or low T1WI and low T2WI signals due to tissue organization. Therefore, bone marrow edema in patients with OVCFs often indicates acute or subacute fractures, which can be effectively treated with PVP to alleviate pain symptoms. On the contrary, PVP is not suitable for the treatment of old vertebral compression fractures without bone marrow edema. Voormolen et al. [20] suggested that the extent of bone marrow edema on preoperative MRI is the only factor associated with pain relief. Thus, bone marrow edema is a useful sign for treatment selection [21]. Moreover, our results also suggest that PVP can alleviate pain symptoms in most patients with a small degree of bone marrow edema. Brown et al. [12] also reported that PVP effectively treats patients without abnormal signals on MRI; however, fluoroscopy revealed a correlation between findings from a physical examination and the compression segment in an imaging study. Nevertheless, we consider a small range of bone marrow edema as an unstable, deformed healing state in the compression body. The mechanism of pain in these patients differs from that in those with acute, subacute, and chronic fractures. The cause of pain might be due to the unstable state of spinal mechanics, the stimulation of Schmorl’s nodes, or other changes in degeneration. Postoperative pain relief might be related to the correction of PVP with regard to the aforementioned factors. This interesting phenomenon should be verified in studies with larger sample sizes.

The advantages of this study are as follows. Because the degree of pain relief differs between local and general anesthesia [22], the latter was selected for all patients to avoid bias caused by different routes of anesthesia. The limitations of this study are listed below. (1) The follow-up visits were scheduled at 1, 6, and 12 months after surgery. The long follow-up intervals might affect the accurate evaluation of the changes in postoperative bone marrow edema and the clinical efficacy. The evaluation might only reflect the approximate trend of the changes. (2) This study is a single-center study with a small sample size.(3) The number of fracture vertebral body was not uniform (One, two, three vertebral bodies). Inconsistency of number will lead to bias from patients.

## Conclusions

PVP is an effective treatment for OVCFs. The MRI characteristics of OVCFs are helpful in predicting their treatment outcomes. Better outcomes were observed among patients with severe or moderate bone marrow edema than among those with mild bone marrow edema. The greater degree of pain relief after PVP was correlated with the faster recovery of postoperative function. However, this correlation gradually became weak over time and disappeared 6 months after surgery. Therefore, PVP should be an option for early stage OVCFs, especially for patients with bone marrow edema signs on MRI.

## Abbreviations

CT: Computed tomography
DSA: Digital subtraction angiography
DXA: Dual-energy X-ray absorptiometry
MOS: Medical outcomes study
MRI: Magnetic resonance imaging
ODI: Oswestry disability index
OVCFs: Osteoporotic vertebral compression fractures
PVP: Percutaneous vertebroplasty
SF-36: 36-Item short-form health survey
SPSS: Statistical product and service solutions
VAS: Visual analogue scale
